# Suggestion as an Aid in Nursing

**Published:** 1920-10-30

**Authors:** R. B. Ince

**Affiliations:** author of "Franz Anton Mesmer: His Life and Teaching"


### SUGGESTION AS AN AID IN NURSING.

By R. B. INCE, author of "Franz Anton Mesmer: His Life and Teaching."

How often does the phrase, "Must be cheerful," or "Cheerful person essential," appear in advertisements for nurses and other social workers? Such a demand is a tacit tribute to the universal recognition of the laws of suggestion.

Everybody knows the adverse effects of the "gloom person" in home or institution. The person with a pessimistic outlook on life never has and never will "get the best" out of others. It is the same with the suspicious person. If you are always suspecting that so-and-so is seeking an opportunity to "do" you, the suspicious habit will increase to your own disadvantage. You will tend to destroy your own peace of mind while destroying that of others. And your faith in the best and the highest will suffer accordingly.

On the other hand, cheerfulness, as even the most ignorant are beginning to understand, is a power in life. Like faith, it can remove mountains if held to firmly and despite all obstacles.

These remarks may appear trite. They are known to all, and frequently insisted upon. But the laws underlying them are not so well known.

Talk to the average person about suggestion, and he, or she, will probably either assume that you are "a bit of a crank" or will betray blank ignorance. Yet suggestion is, and always has been, one of the most potent forces in human life. All of us have, at one time or another, suffered from having some too-popular tune "run," as we say, "in our heads." What is the cause of it? We have heard some catchy tune played over and over again until it has impressed itself on our conscious, and finally on our sub-conscious, minds. Result: Even after we thought we had forgotten it the sub-conscious flings it up into the conscious brain, and the tune returns unbidden to torment us.

If such a condition can result from a chance accident, how much greater must be the influence of the sub-conscious mind when control is taken of it by the will? Modern psychologists are more and more recognising the tremendous possibilities which lie in a strong and reasoned control of the sub-conscious mind.

The nurse who knows something about psychology and the laws of suggestion will always have the advantage of the nurse, no matter how well trained, who is ignorant of these subjects.

A great deal of nonsense has been written about "the will to be well"; and some of the more ardent devotees of the "mind over matter" theory have gone so far as to deny the existence of disease and pain. Such an attitude is as dangerous as it is ridiculous. But out of the froth of talk that surrounds this subject certain broad principles emerge like islands in the trackless ocean.

1. The sub-conscious mind can be influenced through the avenue of the ordinary consciousness. Tell yourself, before falling asleep, and while in that twilight state between waking and sleeping, that you will wake up at a certain hour, and with very little practice your sub-conscious mind will wake you as directed. Suggestions concerning health and character can be given in the same way and will be taken up and acted upon if persisted in.

2. Anyone with a rudimentary knowledge of suggestion can put himself or anyone else to sleep (provided they will co-operate) at any time. This is done by fixing the attention on a point for a few minutes. The nerve-centres grow tired, and sleep results.

3. The mind can be trained just like any other part of the body. Concentration, the induction of sleep, memory, and the conquest of bad habits are all dependent upon a well-trained mind. Life will always be easier, happier, and more interesting for the possessor of a mind that readily obeys the will.

It is well worth while for every nurse who is interested in her profession to make a study of psychology and suggestion.

We have not learnt a great deal since the days when, in 1784, Mesmer astonished the Royal Society of Medicine, of Paris, by his cures. But since then a great change has come over the attitude of the medical faculty towards this subject. We know now that all the functions of the body are controlled by the mind. And that many of the apparently miraculous results of Mesmer and others are due to a working knowledge of the laws of mind.

